# Complex Chaotic Attractor via Fractal Transformation

**DOI:** 10.3390/e21111115

**Published:** 2019-11-14

**Authors:** Shengqiu Dai, Kehui Sun, Shaobo He, Wei Ai

**Affiliations:** School of Physics and Electronics, Central South University, Changsha 410083, China; shengqiu@csu.edu.cn (S.D.); heshaobo_123@163.com (S.H.); aiwei123@csu.edu.cn (W.A.)

**Keywords:** chaos, rotation chaotic attractors, ternary fractal algorithm, dynamics analysis, DSP implementation

## Abstract

Based on simplified Lorenz multiwing and Chua multiscroll chaotic systems, a rotation compound chaotic system is presented via transformation. Based on a binary fractal algorithm, a new ternary fractal algorithm is proposed. In the ternary fractal algorithm, the number of input sequences is extended from 2 to 3, which means the chaotic attractor with fractal transformation can be presented in the three-dimensional space. Taking Lorenz system, rotation Lorenz system and compound chaotic system as the seed chaotic systems, the dynamics of the complex chaotic attractors with fractal transformation are analyzed by means of bifurcation diagram, complexity and power spectrum, and the results show that the chaotic sequences with fractal transformation have higher complexity. As the experimental verification, one kind of complex chaotic attractors is implemented by DSP, and the result is consistent with that of the simulation, which verifies the feasibility of digital circuit implement.

## 1. Introduction

Chaos began in the 20th century, and has such special properties as initial value sensitivity and ergodicity [1,2]. With the development of the chaos theory and its application, it has been investigated extensively in many fields, such as secure communication [3], electronic circuits [4], chemical chaotic system [5], and so on. In the continuous chaotic systems, it is confirmed that the chaotic systems with multiscroll or multiwing exhibit richer dynamics and higher unpredictability [6,7,8,9,10,11]. Therefore, the generation of a multiscroll chaotic system and its circuit implementation are valuable subjects in research. Currently, many design methods are proposed to generate multiwing or multiscroll chaotic system, such as saw-tooth function [12,13,14], hyperbolic tangent function [15,16], hysteresis or saturated sequence [17,18,19], piecewise linear control method [20,21,22], and so on. Among the proposed schemes, it is common that the system equilibrium points are reconstructed by introducing nonlinear functions [23,24,25,26]. However, no matter which nonlinear function is used, the new chaotic system equation is necessary, and the mathematical calculation of the multiscroll or multiwing system will become complicated with the increase in the scrolls. It is an interesting question whether there is a new approach, which can generate multiwing or multiscroll attractors more easily than the traditional approaches.

Fractal transformation is an available method to construct novel chaotic system, and it is applicable for all chaotic system. The core of fractal is self-similarity [27], the same as the chaotic attractor. Actually, a fractal set is a collection of initial points of unstable trajectories in a dynamic system. Therefore, it makes sense to combine chaotic attractors with fractal transformation in consideration of the closely related two disciplines. Meanwhile, the fractal transformation is a good choice for generating multiwing and multiscroll chaotic systems. For example, Guo [28] proposed a fractal transformation map and applied it to a three-dimensional system, but the dynamical performance of this chaotic system has not been significantly changed. A new class of chaotic attractors based on fractal network was proposed by the authors of [29,30]. Bouallegue [31] proposed a new method to generate complex attractors with fractal network. However, they only constructed the fractal chaotic systems without any dynamics analysis, and it can only make fractal transformation with two sequences simultaneously. The dynamical performance of the fractal chaotic systems need to be further analyzed because it is important to select system parameters when the fractal chaotic system is applied to the information security. In addition, the realization of the circuit is significant to the application of chaos. Compared with the analog device, the digital signal processor (DSP) is convenient, stable and reliable for generating chaotic signal [32,33]. Therefore, we intend to implement the fractal chaotic system using the DSP technique.

In this paper, a ternary fractal algorithm is proposed to make the fractal transformation in three-dimensional space, and the fractal algorithm is applied to Lorenz system, rotation Lorenz system and compound chaotic system. To display the changes of chaotic attractors before and after fractal transformation, a comparison between binary fractal and ternary fractal of chaotic system is carried out. The dynamics of the complex chaotic attractors are analyzed by bifurcation diagram, complexity and spectrum distribution. The rest of this paper is organized as follows. The rotation compound chaotic systems are designed in Section 2. The fractal algorithm and its application with chaotic attractors are presented in Section 3. The dynamics of the compound chaotic attractors are analyzed in Section 4. In Section 5, the DSP implementation is presented. Finally, concluding remarks are given.

## 2. The Complex Chaotic Systems Based on Rotation Transformation

### 2.1. Rotation Multiwing Chaotic System

The simplified Lorenz system is defined by [34]

(1) $$\left\{\begin{array}{c} \dot{x}=10\left(y-x\right)\\ \dot{y}=-xz+\left(24-4c\right)x+cy\\ \dot{z}=xy-8/3z \end{array}\right.,$$

where *c* is the system parameter, and the system is chaotic when c∈ (−1.59, 7.75). Applying the rotation transformation to the chaotic system [35], the rotation multiwing chaotic system is obtained as Equation (2). The rotation transformation is a set of mathematical operation rules. One can rotate the attractor to any angle with this operation and the rotation multiwing system is obtained as

(2) $$\left\{\begin{array}{c} \dot{x}=H_{x}\left(H_{1}\cos\left(\theta \right)+H_{3}\sin\left(\theta \right)\right)\\ \dot{y}=H_{2}\\ \dot{z}=H_{z}\left(-H_{1}\sin\left(\theta \right)+H_{3}\cos\left(\theta \right)\right) \end{array}\right.,$$

where $H_{1}$, $H_{2}$, $H_{3}$, $H_{x}$, $H_{z}$, $x^{\prime }$, and $z^{\prime }$ are designed by

(3) $$\begin{array}{c} \left\{\begin{array}{c} H_{1}=100\left(y-0.1x^{\prime }\right)\\ H_{2}=0.1\left(24-4c\right)x^{\prime }-0.1px^{\prime }z^{\prime }+cy\\ H_{3}=F\left(x^{\prime }\right)-8/3z^{\prime }\\ F(x^{\prime })=0.01F_{0}{x^{\prime }}^{2}-\sum_{i=1}^{N}F_{i}\left(1+0.5sgn\left(0.1x^{\prime }-E_{i}\right)-0.5sgn\left(0.1x^{\prime }+E_{i}\right)\right) \end{array}\right.\\ \left\{\begin{array}{c} H_{x}=1/0.0008(|x-x_{0}+0.0004|-|x-x_{0}-0.0004\left|\right)\\ H_{z}=1/0.0008(|z-z_{0}+0.0004|-|z-z_{0}-0.0004\left|\right) \end{array}\right..\\ \left\{\begin{array}{c} z^{\prime }=(|x-x_{0}|+x_{0})\sin\left(\theta \right)+(|z-z_{0}\left|+z_{0}\right)\cos\left(\theta \right)\\ x^{\prime }=(|x-x_{0}|+x_{0})\cos\left(\theta \right)-(|z-z_{0}\left|+z_{0}\right)\sin\left(\theta \right) \end{array}\right. \end{array}$$

**Figure 1:**
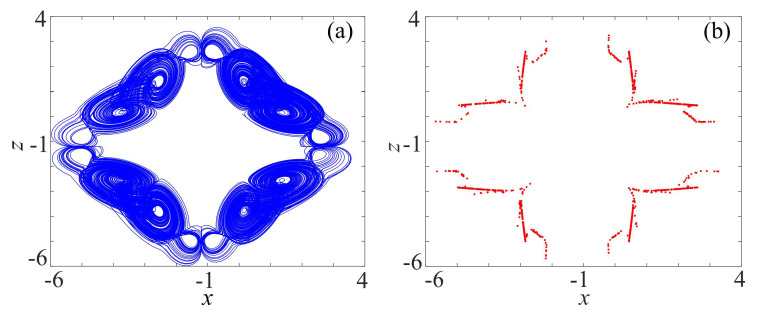
Rotation multiwing chaotic system: (**a**) Phase diagram on x−z plane; and (**b**) Poincaré section (*y* = 0).

It is worth mentioning that θ is the rotation angle, and ($x_{0}$, $z_{0}$) is the central point of the rotation attractor. *p* is a constant, which can facilitate the design and implementation of the corresponding circuits. $F_{0}$ is also an adjustable parameter. The Poincaré section is a good method to characterize chaos. If the Poincaré section is neither a finite point set nor a closed curve, then the system is in a chaotic state. By setting θ = π/4, $x_{0}=-1.2$, $z_{0}=-1.2$, p=20, c=1, $F_{0}=400$, N=1, $F_{1}=20.5$, $E_{1}=0.2$, the initial value is (0.1, 0.1, 2.1), the x−z phase of the rotation attractor diagram and Poincaré section are shown in Figure 1. Obviously, the Poincaré section is consistent with the phase diagram. Calculated by the Wolf algorithm [36], the largest Lyapunov exponent is 2.34, which indicates the rotation multiwing system is chaotic.

### 2.2. Rotation Multiscroll Chaotic System

The Chua chaotic system is described by [37]

(4) $$\left\{\begin{array}{c} \dot{x}=10\left(z-h\left(x\right)\right)\\ \dot{y}=-16z\\ \dot{z}=x-z+y \end{array}\right.,$$

where the piecewise nonlinear function h(x) is defined as

(5) $$\begin{array}{c} h(x)=mx-mn(sgn(x)+sgn(x-2n)+sgn(x+2n)), \end{array}$$

where the parameters *m* and *n* are constants. When h(x) takes a different piecewise linear function, Chua chaotic system can generate different multiscroll attractors [35]. In the experiment, we set *n* = 5, *m* = 0.3 and build a four-scroll Chua chaotic system. Applying the rotation transformation to Equation (4) the rotation Chua chaotic system is obtained as

(6) $$\left\{\begin{array}{c} \dot{x}=\left(3/20\right)S_{x}\left(F_{1}\cos\left(\theta \right)+F_{2}\sin\left(\theta \right)\right)\\ \dot{y}=\left(3/20\right)F_{3}\\ \dot{z}=\left(3/20\right)S_{z}\left(-F_{1}\sin\left(\theta \right)+F_{2}\cos\left(\theta \right)\right) \end{array}\right.,$$

where $F_{1}$, $F_{2}$, $F_{3}$, $S_{x}$, $S_{z}$, $x^{\prime }$, and $z^{\prime }$ are defined by

(7) $$\begin{array}{c} \left\{\begin{array}{c} F_{1}=10\left(x^{\prime }-h\left(z^{\prime }\right)\right)\\ F_{2}=\left(20/3\right)y+z^{\prime }-x^{\prime }\\ F_{3}=-16x^{\prime } \end{array}\right.\\ \left\{\begin{array}{c} S_{x}=1/0.08(|\left(20/3\right)\left(x-1\right)-x_{0}+0.04|-|\left(20/3\right)\left(x-1\right)-x_{0}-0.04\left|\right)\\ S_{z}=1/0.08(|\left(20/3\right)z-z_{0}+0.04|-|\left(20/3\right)z-z_{0}-0.04\left|\right) \end{array}\right..\\ \left\{\begin{array}{c} x^{\prime }=(|\left(20/3\right)\left(x-1\right)-x_{0}|+x_{0})\sin\left(\theta \right)+(|\left(20/3\right)z-z_{0}\left|+z_{0}\right)\cos\left(\theta \right)\\ z^{\prime }=(|\left(20/3\right)\left(x-1\right)-x_{0}|+x_{0})\cos\left(\theta \right)-(|\left(20/3\right)z-z_{0}\left|+z_{0}\right)\sin\left(\theta \right) \end{array}\right. \end{array}$$

**Figure 2:**
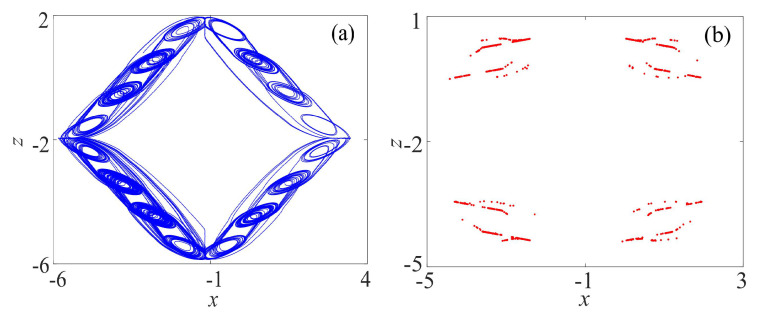
Rotation multiscroll chaotic system: (**a**) Phase diagram on x−z plane; and (**b**) Poincaré section (*y* = 0).

By setting θ = π/4, $x_{0}=-14.5$, $z_{0}=-13$, the initial value is (0.1, 0.1, 0.1), the phase diagram on x−z plane of rotation attractors and the corresponding Poincaré section are shown in Figure 2. The largest Lyapunov exponent is 0.82, which shows that the rotation multiscroll system is chaotic.

### 2.3. Rotation Compound Chaotic System

The compound chaotic attractors have more complex topological structure [6,38,39]. Therefore, to obtain a complicated chaotic system, we construct the compound chaotic system based on rotation multiwing and multiscroll system defined as

(8) $$\left\{\begin{array}{c} \dot{x}=H_{x}\left(H_{1}\cos\left(\theta \right)+H_{3}\sin\left(\theta \right)\right)S_{1}+kS_{x}\left(F_{1}\cos\left(\theta \right)+F_{2}\sin\left(\theta \right)\right)S_{2}\\ \dot{y}=H_{2}S_{1}+kF_{3}S_{2}\\ \dot{z}=H_{z}\left(-H_{1}\sin\left(\theta \right)+H_{3}\cos\left(\theta \right)\right)S_{1}+kS_{z}\left(-F_{1}\sin\left(\theta \right)+F_{2}\cos\left(\theta \right)\right)S_{2} \end{array}\right.,$$

where $F_{1}$, $F_{2}$, $F_{3}$, $H_{1}$, $H_{2}$, and $H_{3}$ are the terms with variable replaced by rotation transformation function. $S_{x}$, $S_{z}$, $H_{x}$, and $H_{z}$ are the piecewise nonlinear functions, and they are the same as above. *k* is the scale factor and $S_{1}$ and $S_{2}$ are the switch controllers, which determine the boundary of multiscroll and multiwing. They are designed by

(9) $$\left\{\begin{array}{c} S_{1}=0.5\left(1+\mathrm{sgn}\left(z-z_{2}\right)\right)\\ S_{2}=0.5\left(1-\mathrm{sgn}\left(z-z_{2}\right)\right) \end{array}\right..$$

**Figure 3:**
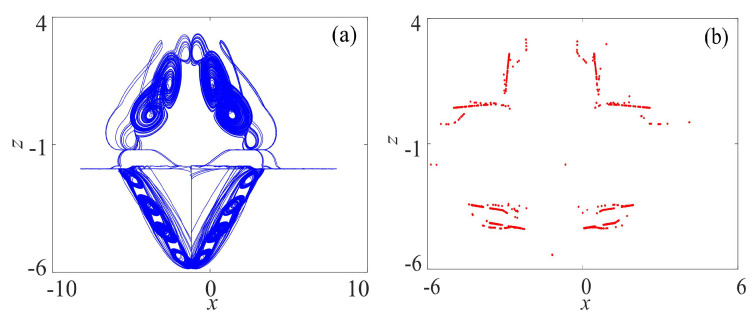
Rotation compound chaotic system: (**a**) Phase diagram on x−z plane; and (**b**) Poincaré section (*y* = 0).

The phase diagram on x−z plane of the compound attractor and the corresponding Poincaré section are shown in Figure 3, and the largest Lyapunov exponent is 2.84, which means the compound rotation chaotic system is chaotic.

## 3. Chaotic Attractors with Fractal Transformation

### 3.1. The Fractal Algorithm

#### 3.1.1. The Binary Fractal Algorithm

The binary fractal algorithm was proposed by Bouallegue [29], but its description is not easy to understand. Thus, we deduce and improve the description of the algorithm. It was based on Julia iterative map, and the map is defined as a quadratic complex Z-map as

(10) $$\begin{array}{c} Z_{n+1}=Z_{n}^{2}+Z_{c}. \end{array}$$

**Figure 4:**
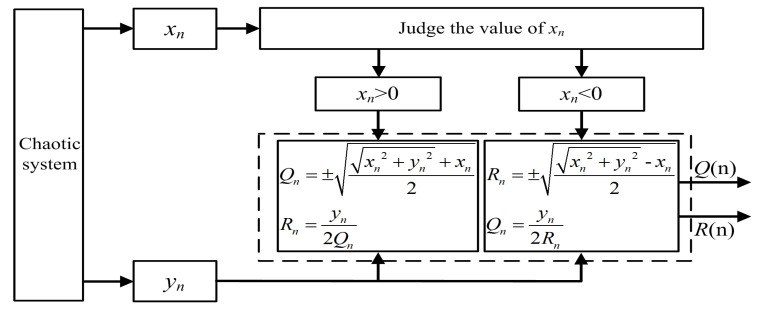
The principle block diagram of the binary fractal algorithm.

If we set $Z_{n}$ = $x_{n}$ + $iy_{n}$, $Z_{n+1}$ = $x_{n+1}$ + $iy_{n+1}$, and $Z_{c}$ = 0, we can obtain $x_{n+1}$ = $x_{n}^{2}$ − $y_{n}^{2}$, $y_{n+1}$=2$x_{n}y_{n}$. Thus, Equation (10) is rewritten as

(11) $$\left\{\begin{array}{c} x_{n+1}=x_{n}^{2}-y_{n}^{2}\\ y_{n+1}=2x_{n}y_{n} \end{array}\right.,$$

and then we exchange the subscripts of variables in Equation (11) to obtain

(12) $$\left\{\begin{array}{c} x_{n}=x_{n+1}^{2}-y_{n+1}^{2}\\ y_{n}=2x_{n+1}y_{n+1} \end{array}\right.,$$

thus $x_{n+1}$ and $y_{n+1}$ are calculated by $x_{n}$ and $y_{n}$ as

(13) $$\left\{\begin{array}{c} x_{n+1}=\pm \sqrt{\frac{\pm \sqrt{x_{n}^{2}+y_{n}^{2}}+x_{n}}{2}}\\ y_{n+1}=\frac{y_{n}}{2x_{n+1}} \end{array}\right.,$$

where $x_{n}$ and $y_{n}$ are the system variables. By evolving Equation (13) into an algorithm, we propose the principle block diagram of the fractal transformation, as shown in Figure 4. The chaotic seed system is solved numerically to the variables $x_{n}$ and $y_{n}$ by the Runge–Kutta method. $x_{n}$ and $y_{n}$ are the input series and $Q_{n}$ and $R_{n}$ are the output series after fractal transformation. The chaotic seed system is the original inputs to the variable $x_{n}$ and $y_{n}$. Then, the variable $x_{n}$ is used as the numerical judgment, and the output series $Q_{n}$ and $R_{n}$ are calculated by the algorithm. This process is called a fractal transformation. Then, the output series $Q_{n}$ and $R_{n}$ can be used as the next input series $x_{n}$ and $y_{n}$, which means the fractal transformation can be looped many times.

#### 3.1.2. The Ternary Fractal Algorithm

The binary fractal algorithm has only two input sequences, and it can only transform two sequences simultaneously. However, many chaotic systems are three-dimensional or higher-dimensional, thus it is significant to construct ternary fractal algorithm. Based on the binary fractal algorithm, we create a new ternary fractal algorithm, which can transform the chaotic system in the three-dimensional space.

Here, we use the ternary number to deduce the ternary fractal algorithm, and the ternary number is described by formula P=a+ib+jc and $i^{2}=j^{2}=-1$. According to Equation (10), we set

(14) $$\left\{\begin{array}{c} Z_{n}=x_{n}+iy_{n}+jz_{n}\\ Z_{n+1}=x_{n+1}+iy_{n+1}+jz_{n+1}\\ Z_{c}=0 \end{array}\right.,$$

and then $Z_{n+1}$ can be calculated by $Z_{n+1}=x_{n}^{2}-y_{n}^{2}-z_{n}^{2}+i2x_{n}y_{n}+j2x_{n}z_{n}+ijy_{n}z_{n}+jiy_{n}z_{n}$. If we set ij=ji=0, then $x_{n+1}=x_{n}^{2}-y_{n}^{2}-z_{n}^{2}$, $y_{n+1}=2x_{n}y_{n}$, and $z_{n+1}=2x_{n}z_{n}$, thus we have

(15) $$\left\{\begin{array}{c} x_{n+1}=x_{n}^{2}-y_{n}^{2}-z_{n}^{2}\\ y_{n+1}=2x_{n}y_{n}\\ z_{n+1}=2x_{n}z_{n} \end{array}\right.,$$

and then we exchange the position of variables in Equation (15) to obtain

(16) $$\left\{\begin{array}{c} x_{n}=x_{n+1}^{2}-y_{n+1}^{2}-z_{n+1}^{2}\\ y_{n}=2x_{n+1}y_{n+1}\\ z_{n}=2x_{n+1}z_{n+1} \end{array}\right.,$$

so the $x_{n+1}$, $y_{n+1}$ and $z_{n+1}$ are calculated by $x_{n}$, $y_{n}$ and $z_{n}$ as

(17) $$\left\{\begin{array}{c} x_{n+1}=\pm \sqrt{\frac{\pm \sqrt{x_{n}^{2}+y_{n}^{2}+z_{n}^{2}}+x_{n}}{2}}\\ y_{n+1}=\frac{y_{n}}{2x_{n+1}}\\ z_{n+1}=\frac{z_{n}}{2x_{n+1}} \end{array}\right..$$

**Figure 5:**
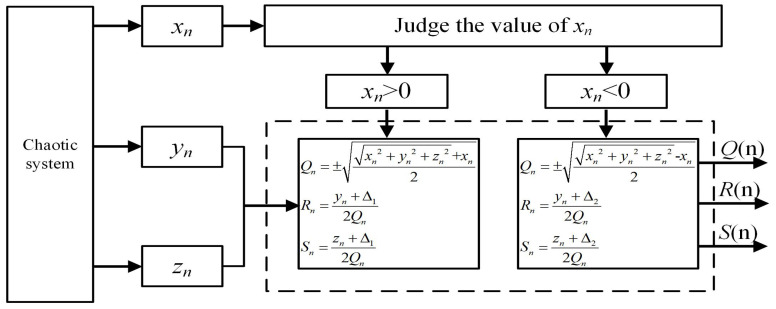
The principle block diagram of the improved fractal algorithm.

To make chaotic attractors more regular after fractal transformation, we modified the algorithm, and the Equation (17) is rewritten as

(18) $$\left\{\begin{array}{c} x_{n+1}=\pm \sqrt{\frac{\pm \sqrt{x_{n}^{2}+y_{n}^{2}+z_{n}^{2}}+x_{n}}{2}}\\ y_{n+1}=\frac{y_{n}+\Delta }{2x_{n+1}}\\ z_{n+1}=\frac{z_{n}+\Delta }{2x_{n+1}} \end{array}\right.,$$

where Δ represents the variable factor, and it is a constant or variable. Here, we set $\Delta_{1}=\Delta_{2}=x_{n}$. To describe ternary fractal transformation clearly, we design the algorithm block diagram, as shown in Figure 5, and it is consistent with the binary one. The chaotic seed system is the original inputs to the variable series $x_{n}$, $y_{n}$ and $z_{n}$. Then, the variable $x_{n}$ is used as the numerical judgment, and the outputs $Q_{n}$, $R_{n}$ and $S_{n}$ are calculated by the algorithm. This process is called a fractal transformation process. The ternary fractal transformation can also be looped many times.

### 3.2. Complex Chaotic Attractors with the Binary Fractal Transformation

#### 3.2.1. Rotation Multiwing with the Binary Fractal Transformation

**Figure 6:**
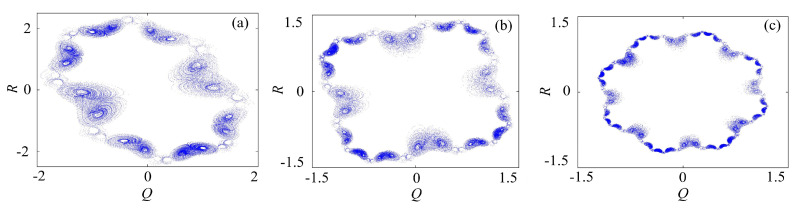
Rotation multiwing chaotic system with the binary fractal transformation: (**a**) once fractal transformation; (**b**) twice fractal transformation; and (**c**) three times fractal transformation.

The binary fractal algorithm is applied to the rotation chaotic system, where *Q* and *R* are the sequences with binary fractal transformation, and the results are shown in Figure 6. It is worth mentioning that, when the input sequence is the original chaotic sequence, we call it the once fractal transformation; when the input sequence is the sequence obtained by the once fractal transformation, we call it the twice fractal transformation; etc. It can be seen in the figure that the wings become 16×2, $16\times 2^{2}$ and $16\times 2^{3}$ after once, twice and three times fractal transformation, respectively, thus we get the relationship between the number of wings and fractal transformation times is exponential. It means that, if the initial number of wings are $M_{1}$, and the number of fractal transformation is *N*, then the number of wings after fractal transformation is $M_{2}=M_{1}\times 2^{N}$.

#### 3.2.2. Rotation Multiscroll with the Binary Fractal Transformation

**Figure 7:**
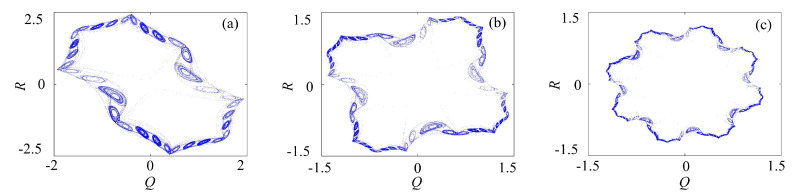
Rotation multiscroll chaotic system with the binary fractal transformation: (**a**) once fractal transformation; (**b**) twice fractal transformation; and (**c**) three times fractal transformation.

The binary fractal algorithm is applied to the rotation multiscroll Chua system, and the results are shown in Figure 7. The *Q* and *R* are the sequences with binary fractal transformation. The relationship between the number of scrolls and fractal transformation times is $M_{2}=M_{1}\times 2^{N}$, where $M_{1}$ is the original number of scrolls, $M_{2}$ is number of scrolls after fractal transformation, and *N* is the transformation times.

#### 3.2.3. Rotation Compound Chaotic System with the Binary Fractal Transformation

**Figure 8:**
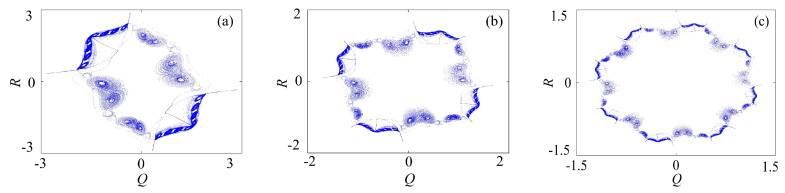
Rotation compound chaotic system with the binary fractal transformation: (**a**) once fractal transformation; (**b**) twice fractal transformation; and (**c**) three times fractal transformation.

The binary fractal algorithm is applied to the rotation compound chaotic system, and the results are shown in Figure 8. *Q* and *R* are the sequence with binary fractal transformation. It also has the same relationship that $M_{2}=M_{1}\times 2^{N}$, where $M_{1}$ is the original number of scrolls and wings, $M_{2}$ is the number of scrolls after fractal transformation, and *N* is the transformation times.

### 3.3. Chaotic Attractors with the Ternary Fractal Transformation

**Figure 9:**
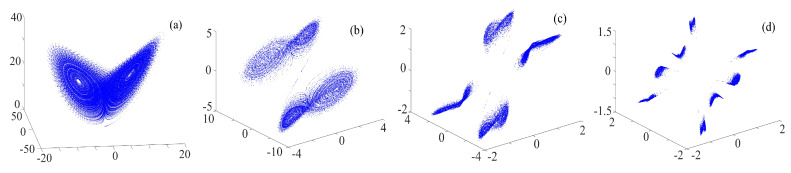
Phase diagram of the Lorenz system with the ternary fractal transformation: (**a**) Lorenz system; (**b**) once fractal transformation; (**c**) twice fractal transformation; and (**d**) three times fractal transformation.

To display the ternary fractal transformation more clearly, we choose the simple chaotic system such as Lorenz system to do the transformation. $x_{0}=1$, $y_{0}=2$, and $z_{0}=3$ are set, and the results are shown in Figure 9. It is clear that the chaotic attractors with ternary fractal transformation are symmetrically distributed in the space.

**Figure 10:**
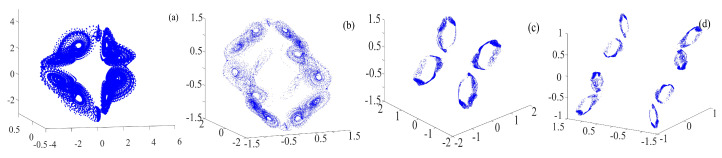
Rotation Lorenz system with the ternary fractal transformation: (**a**) rotation Lorenz system; (**b**) once fractal transformation; (**c**) twice fractal transformation; and (**d**) three times fractal transformation.

**Figure 11:**
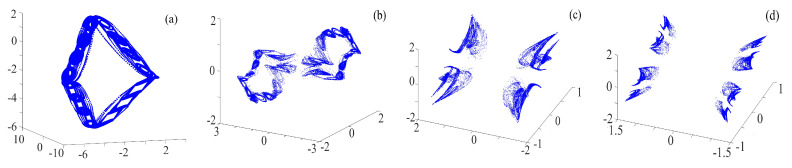
Rotation Chua system with the ternary fractal transformation: (**a**) rotation Chua system; (**b**) once fractal transformation; (**c**) twice fractal transformation; and (**d**) three times fractal transformation.

**Figure 12:**
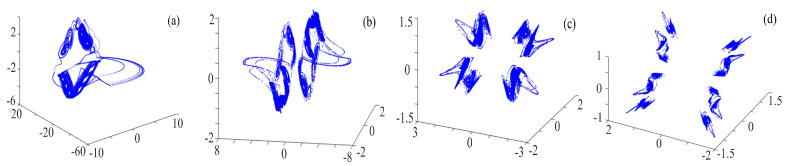
Compound rotation chaotic system with the ternary fractal transformation: (**a**) rotation compound chaotic system; (**b**) once fractal transformation; (**c**) twice fractal transformation; and (**d**) three times fractal transformation.

The results of setting θ = π/4, $x_{0}=0.1$, $y_{0}=0.1$, and $z_{0}=0.1$ and applying the ternary fractal transformation to the rotation chaotic system are shown in Figure 10, Figure 11 and Figure 12. As can be seen in the figures, the number of rings with ternary fractal transformation is exponentially related to the number of fractal transformation, which means, if $M_{1}$ is the original number of rings, $M_{2}$ is the number of rings after fractal transformation, and *N* is the transformation times, then $M_{2}=M_{1}\times 2^{N}$. It is the same as binary fractal transformation.

## 4. Dynamics Analysis of the Complex Chaotic Systems

The dynamics analysis is important for the application, and here we choose the bifurcation diagram, complexity and spectrum distribution to measure the characteristics of chaotic systems.

### 4.1. Bifurcation Diagram

**Figure 13:**
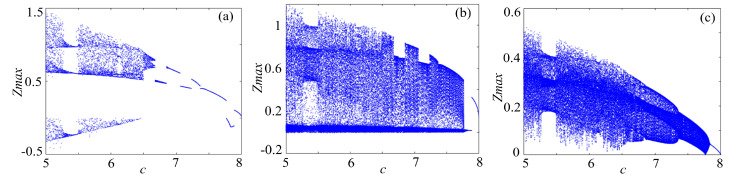
Bifurcations of the Lorenz system: (**a**) Lorenz system; (**b**) Lorenz system with binary fractal transformation; and (**c**) Lorenz system with ternary fractal transformation.

**Figure 14:**
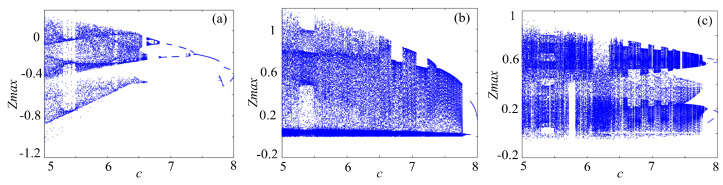
Bifurcations of the rotation Lorenz system: (**a**) rotation Lorenz system; (**b**) rotation Lorenz system with binary fractal transformation; and (**c**) rotation Lorenz system with ternary fractal transformation.

**Figure 15:**
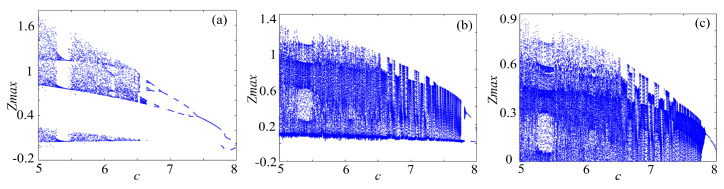
Bifurcations of the compound chaotic system: (**a**) compound chaotic system; (**b**) compound chaotic system with binary fractal transformation; and (**c**) compound chaotic system with ternary fractal transformation.

Bifucation diagram is a significant indicator to evaluate the dynamical characteristics of a chaotic system. To make a clear comparison, the bifurcation of rotation chaotic systems before and after fractal transformation are calculated, and the results are shown in Figure 13, Figure 14 and Figure 15. It is clear that there are some periodic windows in the chaotic attractor before fractal transformation, but many of the periodic windows disappear after fractal transformation, which means the fractal transformation expands the parameter range of chaotic state.

### 4.2. Complexity Analysis

There are kinds of methods to measure the complexity, such as spectrum entropy (SE), C_0_ entropy, and permutation entropy (PE) [40,41,42]. Among them, PE algorithm is a proper choice to estimate the numerical series accurately and rapidly. Thus, the complexity of the complex chaotic system with fractal is analyzed by permutation entropy (PE) algorithm. The larger the PE value is, the more complex the time series are.

**Figure 16:**
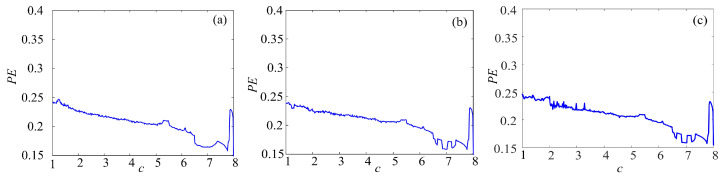
PE complexity of chaotic system: (**a**) Lorenz system; (**b**) rotation Lorenz system; and (**c**) compound chaotic system.

**Figure 17:**
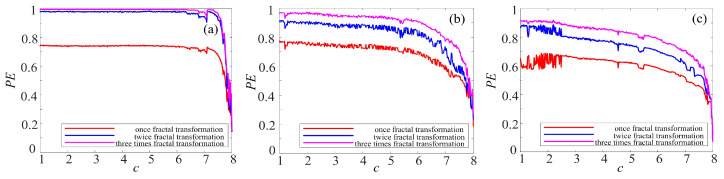
PE complexity of chaotic system with fractal transformation: (**a**) Lorenz system with the binary fractal transformation; (**b**) rotation Lorenz system with binary fractal transformation; and (**c**) compound chaotic system with binary fractal transformation.

**Figure 18:**
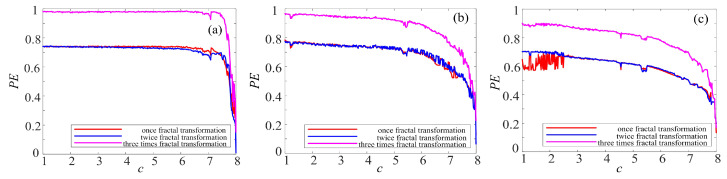
PE complexity of chaotic system with the fractal transformation: (**a**) Lorenz system with ternary fractal transformation; (**b**) rotation Lorenz system with ternary fractal transformation; and (**c**) compound chaotic system with ternary fractal transformation.

To make better comparison, the PE complexity of the original chaotic system before and after fractal transformation are calculated. The results are shown in Figure 16, Figure 17 and Figure 18. It is easy to see in Figure 16 that the PE of Lorenz system and rotation Lorenz system are similar, and it is at a lower level. In Figure 17a, the complexity of rotation Lorenz system after once binary fractal transformation increases from about 0.24 to 0.75, and it increases from about 0.75 to 0.98 after twice fractal transformation. After three times fractal transformation, it reaches 0.99. In Figure 17b, the complexity of the rotation Lorenz after once fractal transformation increases from about 0.24 to about 0.77, it increases from about 0.77 to around 0.9 after twice fractal transformation, and it increases from about 0.9 to 0.95 after three times fractal transformation. In Figure 17c, we can obtain that the PE increases with the number of fractal transformation. In Figure 18, we also observe the complexity increases greatly after ternary fractal transformation. Obviously, compared with the original system, the binary and ternary fractal transformation can both improve the complexity of chaotic systems.

### 4.3. Spectrum Distribution Characteristics

**Figure 19:**
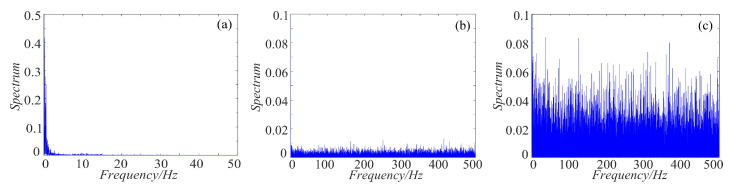
Spectrum distribution of the rotation Lorenz system with fractal transformation: (**a**) rotation Lorenz system; (**b**) rotation Lorenz system with binary fractal transformation; and (**c**) rotation Lorenz system with ternary fractal transformation.

**Figure 20:**
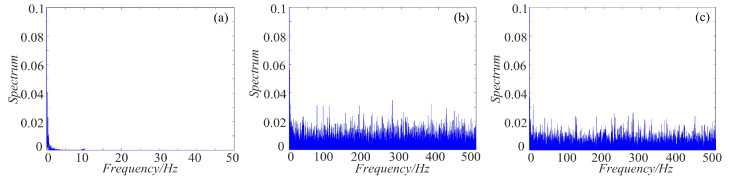
Spectrum distribution of the rotation Chua system with fractal transformation: (**a**) rotation Chua system; (**b**) rotation Chua system with binary fractal transformation; and (**c**) rotation Chua system with ternary fractal transformation.

**Figure 21:**
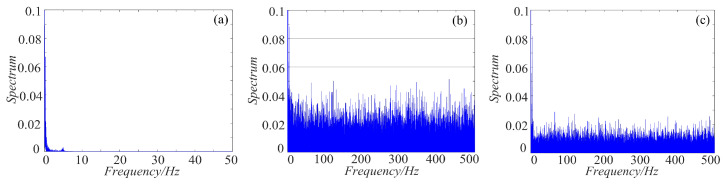
Spectrum distribution of the compound chaotic system with fractal transformation: (**a**) compound chaotic system; (**b**) compound chaotic system with binary fractal transformation; and (**c**) compound chaotic system with ternary fractal transformation.

As we all know, the power spectrum of the period signal is a discrete spectrum and the power spectrum of the non-periodic signal is a continuous spectrum. Chaos signal is non-periodic, thus it is continuous. For non-periodic signal, the more uniform the spectrum is, the more complex the signal is. To make a clear comparison, the spectrum distribution of rotation chaotic systems before and after fractal transformation are calculated, and the results are shown in Figure 19, Figure 20 and Figure 21. As shown in Figure 19, the power spectrum of the rotation Lorenz system before fractal transformation is distributed in the range of 0–5 Hz. After binary and ternary fractal transformation, the power spectrum of the chaotic system is distributed uniformly in 0–500 Hz, which shows the complexity of the system is improved after fractal transformation. In the Figure 20 and Figure 21, the rotation Chua and compound chaotic system have the same phenomenon with the rotation Lorenz system. Obviously, compared with the original system, the binary and ternary fractal transformation can also improve the distribution characteristics of chaotic systems.

## 5. DSP Implementation

**Figure 22:**
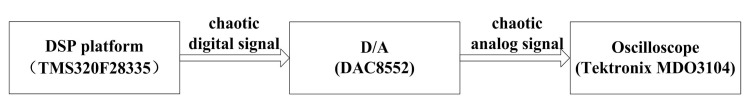
Flow diagram of the DSP implementation.

The digital circuit of the fractal chaotic system is implemented based on DSP technique. There are three parts, as shown in Figure 22. The key chip is DSP TMS320F28335, and the D/A converter DAC8552 is a 16-bit dual-channel converter. The calculations are carried out on the DSP platform. The signals are converted from digital signals into analog signals via D/A converter, and then the analog signals are sent to oscilloscope, which is used to record phase portraits of the system.

**Figure 23:**
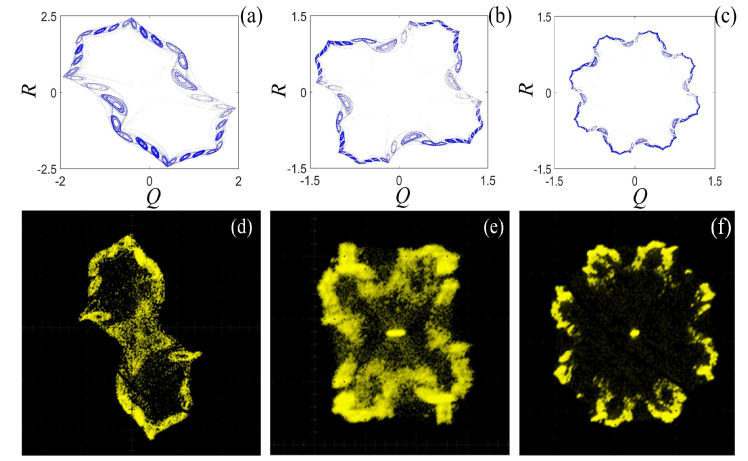
Computer simulation results of the rotation Chua chaotic system: (**a**) once fractal transformation; (**b**) twice fractal transformation; and (**c**) three times fractal transformation. DSP results of the rotation Chua chaotic system: (**d**) once fractal transformation; (**e**) twice fractal transformation; and (**f**) three times fractal transformation.

Based on the the rotation Chua chaotic system system in Equation (6) and the binary fractal algorithm in Equation (13), we set the same values of the system parameters in DSP experiment, and compare the computer simulation results with the DSP results. The phase diagrams of the rotation Chua system after fractal transformation are shown in Figure 23. Obviously, the computer simulation results in Figure 23a–c are consistent with the DSP results in Figure 23d–f. It is worth mentioning that the differential equations are discretized by employing modified Euler method. The computational precision of DSP program is different from the computer simulation, which employs the fourth-order Runge–Kutta method, thus the distribution uniformity of attractor shown in oscilloscope is a little bit different from the computer simulation.

## 6. Conclusions

In this paper, a novel ternary fractal algorithm is proposed, and the complex chaotic attractors are generated by employing fractal transformation. The dynamics of the complex chaotic systems with fractal transformation are analyzed by bifurcation, PE complexity and spectrum distribution. The results show that whether simple or complex chaotic system, and whether binary or ternary fractal transformation, the PE complexity increases greatly after fractal transformation. The complexity of a chaotic system with binary fractal transformation increases with the number of fractal transformation, until it approaches infinitely close to 1, and the number of wings or scrolls of chaotic attractors is exponentially related to the fractal transformation times. The DSP experiments show a good agreement with computer simulation. More complex topological structure of chaotic systems could be generated by modified fractal algorithm. It has potential applications in the future.
